# Bioaccessibility *In Vitro* of Nutraceuticals from Bark of Selected *Salix* Species

**DOI:** 10.1155/2014/782763

**Published:** 2014-02-17

**Authors:** Urszula Gawlik-Dziki, Danuta Sugier, Dariusz Dziki, Piotr Sugier

**Affiliations:** ^1^Department of Biochemistry and Food Chemistry, University of Life Sciences, Skromna Street 8, 20-704 Lublin, Poland; ^2^Department of Industrial and Medicinal Plants, University of Life Sciences in Lublin, Akademicka 15, 20-950 Lublin, Poland; ^3^Department of Thermal Technology, University of Life Sciences, Doświadczalna 44, 20-280 Lublin, Poland; ^4^Department of Ecology, Faculty of Biology and Biotechnology, Maria Curie-Skłodowska University, Akademicka 19, 20-033 Lublin, Poland

## Abstract

The aim of this study was to investigate and to compare the extractability, bioaccessibility, and bioavailability *in vitro* of antioxidative compounds from bark of selected *Salix* species: *S. alba (SA), S. daphnoides (SD), S. purpurea (SP),* and *S. daphnoides x purpurea (SDP)* hybrid willow clones originating from their natural habitats and cultivated on the sandy soil. The highest amount of phenolic glycosides was found in the bark of *SDP* and *SD*. The best source of phenolics was bark of *SDP*. The highest content of flavonoids were found in *SD* bark samples, whereas the highest concentration of bioaccessible and bioavailable phenolic acids was determined in *SDP* bark. Bark of all tested *Salix* species showed significant antiradical activity. This properties are strongly dependent on extraction system and genetic factors. Regardless of *Salix* genotypes, the lowest chelating power was found for chemically-extractable compounds. Bark of all *Salix* species contained ethanol-extractable compounds with reducing ability. Besides this, high bioaccessibility and bioavailability *in vitro* of *Salix* bark phytochemicals were found. Obtained results indicate that extracts from bark tested *Salix* genotypes can provide health promoting benefits to the consumers; however, this problem requires further study.

## 1. Introduction

The willow bark is a constituent of many herbal drugs and also dietary supplements such as an analgesic, antipyretic, antiphlogistic and weight loss enhancement remedies. The term *Salicis Cortex* [SC] is defined as whole or fragmented dried bark of young branches or whole dried pieces of current year twigs of various species of the genus *Salix* [1]. SC is standardised based on the content of salicin, a compound with analgesic and antiphlogistic properties. However, clinical trials suggest that other compounds also present in Salicis Cortex can contribute to the pharmacological effects. The results of clinical trials suggest that, besides salicylic derivatives, other substances like polyphenols (flavonoids, flavan-3-ols) and simple phenols (phenolic acids) can contribute to the therapeutic effects of SC [2, 3].

Phenolic compounds possess strong antioxidant activity; thus, this effect probably results from synergistic interactions between them and salicin. Antioxidant activity is the fundamental property of phenolic medicinal plant compounds important for their health protecting, including antimutagenic, anticarcinogenic, and antiaging activity. Reactive oxygen species (ROS) scavenging preserves the genomic stability of cells through elimination of carcinogens and interference with DNA adducts formation [4]. Free radicals are constantly generated in the body as a result of oxidative metabolism. The creation of ROS also is connected with lipoxygenase (LOX) and xanthine oxidase (XO) activity. Subsequently, oxidative stress occurs if the antioxidant defense in the organism is not adequate. The condition of *in vivo* “oxidative stress” is defined as elevated levels of free radicals or other ROS which can elicit either direct or indirect damage to the body [5].

Plant extracts containing secondary metabolites have served as antioxidants in phytotherapeutic medicines to protect against various diseases for centuries. Natural antioxidants exhibit a wide range of pharmacological activities and have been shown to have anticancer, anti-inflammatory and antiaging properties [6]. Accordingly, phytochemicals can directly interfere with signaling systems involved in the regulation of inflammatory processes, angiogenesis and cancer invasion in a manner dependent on their antioxidative activity and concomitant inhibitory effect on the enzymes involved with pathological condition [7, 8].

*S. purpurea *L.*, S. daphnoides *Vill., and *S. alba* L. are a very popular herbal species affirmed in the natural habitats and field-cultivated in Poland [9, 10]. The phenolic glycosides contained in willow bark of this species are known for their anti-inflammatory, analgesic, and fever-reducing effects and have been shown to relieve rheumatic disturbances, infections, and headache [3, 11]. Additionally, bark of this species contains *p*-hydroxybenzoic, vanillic, cinnamic, *p*-coumaric, ferulic, and caffeic acids phenolic acids and naringenin [1]. However little is as yet known about their synergic effects. A particularly important issue on the effectiveness of dietary supplements is bioavailability and bioavailability of active compounds. Thus the aim of this study was to investigate and to compare the extractability, bioaccessibility, and bioavailability *in vitro* of antioxidative compounds from bark of selected *Salix* species.

## 2. Materials and Methods 

### 2.1. Plant Material

Selected *S. alba (SA), S. daphnoides (SD), S. purpurea (SP),* and *S. daphnoides x purpurea (SDP)* hybrid willow clones with the highest phenolic glycoside contents [9, 10] originating from their natural habitats were cultivated on the sandy soil (heavy loamy sand). The study was carried out in 2009 and 2010 on a 5-6 year-old plantation. The experiment was a completely randomized block design with three replicates conducted in the area of experimental fields at the University of Life Sciences in Lublin (51°33′N; 22°44′E). The plantation was established at the spacing of 40 × 20 cm. Each plot was 16 m^2^. The sandy soil was characterized by an average content of humus (1.41%), very low phosphorus (17.3 mg·kg^−1^), very low potassium (33.2 mg·kg^−1^), very low magnesium (15.0 mg·kg^−1^), and strong acid reaction (pH KCl—4.1). Mineral fertilization was applied in spring before the beginning of vegetation in both soil types: N—20 kg; P—13.1 kg; K—49.8 kg calculated per 1 ha.

Willow shoots were harvested in November every year (2009-2010) in three replicates. The plant material was collected in the form of 60 annual shoots (20 entire shoots per plot) from every taxon. The shoots were washed with deionized water. Bark was separated from the wood by peeling and subsampled for chemical analysis.

The bark material sampled for salicylate analysis was dried at room temperature and intensively mixed and homogenized. After drying, the phenolic glycosides content calculated on salicin was determined by means of the HPLC technique [12] in the Laboratory of Labofarm in Starogard Gdański and expressed as mg/mg dry mass (DM).

### 2.2. Chemicals

Ferrozine (3-(2-pyridyl)-5,6-bis-(4-phenyl-sulfonic acid)-1,2,4-triazine), ABTS (2,2′-azino-bis (3-ethylbenzothiazoline-6-sulphonic acid)) *α*-amylase, pancreatin, pepsin, bile extract, Folin-Ciocalteau reagent, linoleic acid, ammonium thiocyanate, and haemoglobin were purchased from Sigma-Aldrich Company (Poznan, Poland). All others chemicals were of analytical grade.

### 2.3. Extracts Preparation

#### 2.3.1. Chemical Extract (CE)

Two grams of plant material was filled with 100 mL of 70% ethanol and left in darkness by 2 weeks.

#### 2.3.2. Buffer Extract (BE)

Powdered samples of willow bark (1 g) were extracted for 1 h with 20 mL of PBS buffer (phosphate buffered saline, pH 7.4). The extracts were separated by decantation and the residues were extracted again with 20 mL of PBS buffer. Extracts were combined and stored in darkness at −20°C.

#### 2.3.3. Digestion *In Vitro* (DE)

*In vitro* digestion and absorption were performed according to Gawlik-Dziki [13]. The bark samples (1 g) were homogenized in a stomacher laboratory blender for 1 min to simulate mastication with the presence of 15 mL of simulated salivary fluid (prepared by dissolving 2.38 g Na_2_HPO_4_, 0.19 g KH_2_PO_4_, and 8 g NaCl, 100 mg of mucin in 1 liter of distilled water. The solution was adjusted to pH = 6.75 and *α*-amylase (E.C. 3.2.1.1.) was added to obtain 200 U per mL of enzyme activity. For the gastric digestion 300 U/mL of pepsin (from porcine stomach mucosa, pepsin A, EC 3.4.23.1) in 0.03 mol/L NaCl, pH = 1.2 was prepared. Further, simulated intestinal juice was prepared by dissolving 0.05 g of pancreatin (activity equivalent 4 x USP) and 0.3 g of bile extract in 35 mL 0.1 mol/L NaHCO_3_), and, subsequently, the samples were shaken for 10 min at 37°C. The samples were adjusted to pH = 1.2 using 5 mol/L HCl, and, subsequently, 15 mL of simulated gastric fluid was added. The samples were shaken for 60 min at 37°C. After digestion with the gastric fluid, the samples were adjusted to pH = 6 with 0.1 mol/L of NaHCO_3_ and then 15 mL of a mixture of bile extract and pancreatin was added. The extracts were adjusted to pH = 7 with 1 mol/L NaOH and finally 5 mL of 120 mmol/L NaCl and 5 mL of mmol/L KCl were added to each sample. The prepared samples were submitted for *in vitro* digestion for 120 minutes, at 37°C in the darkness. After that, samples were centrifuged and supernatants were used for further analysis.

#### 2.3.4. Absorption *In Vitro* (AE)

Considering that antioxidants absorption takes place mainly at the intestinal digestion stage, the resulting mixture (fluids obtained after *in vitro* digestion) was transferred to the dialysis sacks (D9777-100FT, Sigma-Aldrich), placed in an Erlenmeyer flask containing 50 mL of PBS buffer and incubated in a rotary shaker (2 times per 2 h, 37°C). The PBS buffer, together with the compounds that passed through the membrane (dialysate) was treated as an equivalent of the raw material absorbed in the intestine after digestion.

### 2.4. Determination of Total Phenolics Content (TPC)

Total phenols were estimated according to the Folin-Ciocalteau method [14]. A 0.5 mL sample of the extract was mixed with 0.5 mL of H_2_O, 2 mL of Folin reagent (1 : 5 H_2_O), and after 3 min with 10 mL of 10% Na_2_CO_3_. After 30 min, the absorbance of mixed samples was measured at a wavelength of 725 nm. The amount of total phenolics was expressed as gallic acid equivalents (GAE) per gram of dry mass (DM).

### 2.5. Determination of Total Flavonoids (TFC)

Total flavonoids were estimated according to the method described by Bahorun et al. [15]. One milliliter of sample was mixed with 1 mL 2 g/100 mL, AlCl_3_  ×  6H_2_O. After 10 min absorbance at 430 nm was measured. The total flavonoids content was expresses as quercetin equivalent (QE) in milligrams per gram of DM.

### 2.6. Determination of Total Phenolic Acids (TPA)

Total phenolic acids content was determined according to the Arnov method [16]. One milliliter of sample was mixed with 5 mL of distilled water, 1 mL 0.5 mol/L HCl, 1 mL of Arnov reagent (10 g sodium molybdate and 10 g sodium nitrite dissolved in 100 mL of distilled water) and 1 mL 1 mol/L NaOH and complete to 10 mL with distilled water. Absorbance was measured at 490 nm. The total phenolic acids content was expressed as caffeic acid equivalent (CAE) in micrograms per gram of DM.

### 2.7. Free Radical Scavenging Assay (AA)

The experiments were performed using an improved ABTS decolorization assay [17]. ABTS^+•^ was generated by the oxidation of ABTS with potassium persulfate. The ABTS radical cation (ABTS^+•^) was produced by reacting 7 mmol/L stock solution of ABTS with 2.45 mmol/L potassium persulphate (final concentration). The ABTS^+•^ solution was diluted (with distilled water) to an absorbance of 0.7 ± 0.05 at 734 nm. Then, 40 *μ*L of sample was added to 1.8 mL of ABTS^+•^ solution and the absorbance was measured at the end time of 5 min. The ability of the extracts to quench the ABTS free radical was determined using the following equation:

(1) $$\begin{array}{c} \text{scavenging}\;\%=\left[\frac{\left(A_{C}-A_{A}\right)}{A_{C}}\right]\times 100, \end{array}$$

where *A*_*C*_: absorbance of control and *A*_*A*_: absorbance of sample.

Antioxidant activity was expressed as IC_50_—extract concentration provided 50% of activity based on dose-dependent mode of action.

### 2.8. Metal Chelating Activity (CHEL)

Chelating power was determined by the method of Guo et al. [18]. The extract samples (5 mL) were added to a 0.1 mL of 2 mM FeCl_2_ solution and 0.2 mL 5 mM ferrozine and the mixture was shaken vigorously and left standing at room temperature for 10 min. Absorbance of the solution was then measured spectrophotometrically at 562 nm. The percentage of inhibition of ferrozine-Fe^2+^ complex formation was given below formula:

(2) $$\begin{array}{c} \%\;\text{inhibition}=\left[1-\left(\frac{A_{P}}{A_{C}}\right)\right]\times 100, \end{array}$$

where *A*_*C*_: absorbance of the control and *A*_*P*_: absorbance in the presence of the sample.

Antioxidant activity was expressed as IC_50_—extract concentration provided 50% of activity based on dose-dependent mode of action.

### 2.9. Ferric Reducing Power (FRAP)

Reducing power was determined using the method described by Oyaizu [19]. Extracts (2.5 mL) were mixed with phosphate buffer (2.5 mL, 200 mmol/L, pH 6.6) and 2.5 mL of 1 g/100 mL aqueous solution of potassium ferricyanide K_3_[Fe(CN_6_)]. The mixture was incubated at 50°C for 20 min. A portion (0.5 mL) of 10 g/100 mL trichloroacetic acid was added to the mixture, which was then centrifuged at 25 ×g for 10 min. The upper layer of solution (2.5 mL) was mixed with distilled water (2.5 mL) and 0.5 mL of 0.1 g/100 mL FeCl_3_, and the absorbance was measured at 700 nm. IC_50_ value (mg/mL) is the effective concentration at which the absorbance was 0.5 for reducing power and was obtained by interpolation from linear regression analysis.

### 2.10. Inhibition of Lipoxygenase (LOXI)

Lipoxygenase activity was determined spectrophotometrically at a temperature of 25°C by measuring the increase of absorbance at 234 nm over a 2 min period [20]. The reaction mixture contained 2.45 mL 1/15 mol/L phosphate buffer, 0.02 mL of lipoxygenase solution (167 U/mL), and 0.05 mL of inhibitor (vegetable extract) solution. After preincubation of the mixture at 30°C for 10 min, the reaction was initiated by adding 0.08 mL 2.5 mmol/L linoleic acid. One unit of LOX activity was defined as an increase in absorbance of 0.001 per minute at 234 nm.

Antioxidant activity was expressed as IC_50_—extract concentration provided 50% of activity based on dose-dependent mode of action.

### 2.11. Inhibition of Xanthine Oxidase (XOI)

The XOI activities with xanthine as a substrate were measured spectrophotometrically [21], with the following modification: the assay mixture consisted of 0.5 mL of test solution, 1.3 mL of 1/15 mol/L phosphate buffer (pH 7.5), and 0.2 mL of enzyme solution (0.01 U/mL in M/15 phosphate buffer). After preincubation of the mixture at 30°C for 10 min, the reaction was initiated by adding 1.5 mL of 0.15 mmol/L xanthine solution. The assay mixture was incubated at 30°C and the absorbance (295 nm) was measured every minute for 10 min. XO inhibitory activity was expressed as the percentage inhibition of XO in the above assay mixture system and was calculated as follows:

(3) $$\begin{array}{c} \%\;\text{inhibition}=\left(1-\frac{\Delta A/\min_{\text{test}}}{\Delta A/\min_{\text{blank}}}\right)\times 100, \end{array}$$

where Δ*A*/min⁡_test_ is the linear change in absorbance per minute of test material and Δ*A*/min⁡_blank_ is the linear change in absorbance per minute of blank.

Antioxidant activity was expressed as IC_50_—extract concentration provided 50% of activity based on dose-dependent mode of action.

### 2.12. Theoretical Approach

The following factors were determined to better evaluate the extractability of phenolic compounds:

(i) the mastication efficiency factor (MEF) which is an indication extractability of the phytochemicals during simulated mastication
  (4) $$\begin{array}{c} \text{MEF}=\frac{C_{\text{BE}}}{C_{\text{CE}}}\;, \end{array}$$
(ii) the digestion efficiency factor (DEF) which is an indication extractability of the phytochemicals during simulated digestion
  (5) $$\begin{array}{c} \text{DEF}=\frac{C_{\text{GE}}}{C_{\text{CE}}}, \end{array}$$
(iii) the absorption efficiency factor (AEF) which is an indication extractability of the phytochemicals during simulated absorption
  (6) $$\begin{array}{c} \text{AEF}=\frac{C_{\text{AE}}}{C_{\text{CE}}}, \end{array}$$
  where *C*_BE_ is the concentration of phenolics in raw extract (BE), *C*_GE_ is the concentration of phenolics in extract after simulated gastrointestinal digestion (GE), and *C*_AE_ is the concentration of phenolics in extract after simulated intestinal absorption (AE).

The following factors were determined to better understand the relationships between biologically active compounds in the light of their bioaccessibility, bioavailability, and bioefficiency:

(i) the antioxidant bioaccessibility index (BAC), which is an indication of the bioaccessibility of antioxidative compounds:
  (7) $$\begin{array}{c} \text{BAC}=\frac{A_{\text{BE}}}{A_{\text{GE}}}, \end{array}$$
(ii) the antioxidant bioavailability index (BAV) which is an indication of the bioavailability of antioxidative compounds:
  (8) $$\begin{array}{c} \text{BAV}=\frac{A_{\text{GE}}}{A_{\text{AE}}}, \end{array}$$
(iii) the antioxidant bioefficiency index (BEF), which is an indication of the bioactivity of bioavailable antioxidant compounds:
  (9) $$\begin{array}{c} \text{BEF}=\frac{A_{\text{BE}}}{A_{\text{AE}}}, \end{array}$$
  where *A*_BE_ is EC_50_ of raw extract (BE), *A*_GE_ is IC_50_ of extract after simulated gastrointestinal digestion (GE), and AAE is IC_50_ of extract after simulated intestinal absorption (AE).

### 2.13. Statistical Analysis

All experimental results are displayed as ± S.D. of three parallel experiments (*n* = 9) and data were evaluated by analysis of variance (one-way Anova). The statistical differences between the groups were estimated using the Tukey test, *α* = 0.05.

## 3. Results and Discussion

### 3.1. Comparison of Phytochemicals Content

**Figure 1:**
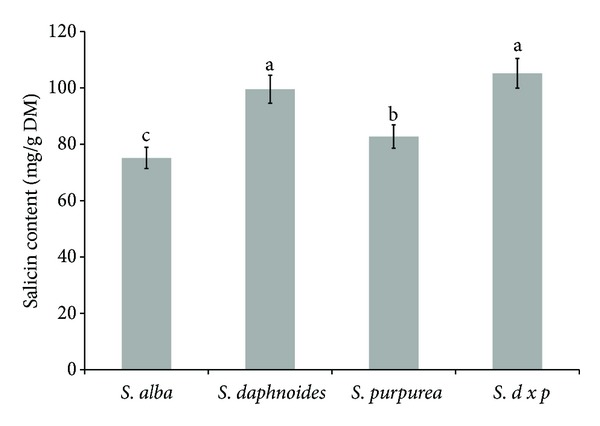
Comparison of phenolic glycosides content in *Salix* bark. Bars (means) followed by the different letters differ significantly (Tukey-test, *P* < 0.05).

*Salix* bark samples were characterized by a diverse content of phenolic glycosides. The highest amounts were found in the bark of *SDP* and *SD*, whereas the lowest—in the bark of *SA*  (Figure 1). These results were in accordance with those obtained by Sugier et al. [10]. Several studies have focused on phytochemical investigation of *Salix* species used for preparing the final willow bark products, and, for example, *S. daphnoides*, *S. pentandra*, *S. purpurea*, *S. alba*, and *S. fragilis* have been investigated for their content of phenolic glycosides. Salicylates (calculated as salicin) are found in all members of *Salix* species but *S. daphnoides*, *S. fragilis*, and *S. purpurea* contain the greatest yield [22]. These constituents are also reported to possess antirheumatic, antipyretic, hyperglycemic/hypoglycemic, uricosuric/antiuricosuric activities, increases prothrombin time, and plasma-albumin binding [23]. In addition to salicylates, flavonoids and condensed tannins constitute the major groups of secondary metabolites in *Salix* species, and these compounds are believed to contribute to the analgesic and anti-inflammatory effect of willow bark [24].

As it can be seen from Table 1, regardless of kind of extract, the best source of phenolic compounds (expressed as gallic acid equivalent) was bark of *SDP*. However, the highest contents of flavonoids were found in samples obtained from *SD* bark. Taking into account alcohol and buffer extracts, the highest content of phenolic acids was determined in samples obtained from *SD* bark; however the highest their concentration was determined in extracts obtained after simulated digestion and absorption of *SDP* bark. Taking into account data presented in Table 1 it can be concluded that the least valuable source of phenolic compounds was *SA* bark.

Taking into account extractability of phenolic compounds fractions, the highest extractability of total phenolics in all samples has been reached in the simulated mastication stage, whereas simulated digestion efficiency was significantly lower. Importantly, efficiency of simulated absorption (with the exception of *SA* bark) was comparable to that determined for the simulated digestion system. These results may indicate high bioavailability of phenolic compounds from *Salix* bark. Taking into account flavonoids and phenolic acids, the highest efficiency was found for simulated digestion system (except *SA* phenolic acids), which may indicate high bioaccessibility of these compounds. For flavonoids, effectiveness of simulated absorption system was the lowest, which may indicate low bioavailability of these compounds, whereas for phenolic acids, AEF values were generally comparable to DEF values, which may indicate high bioavailability of phenolic acids from tested sources (Table 1).

Results concerning total phenolics content obtained in our laboratory were significantly higher than those concerning *S. aegyptiaca* [25]. The total phenol content of 212 ± 4 mg GAE/g of DM was obtained in the ethanolic extract of S. *aegyptiaca* bark while the lowest total phenol content of 4 ± 1 mg GAE/g of dried sample was obtained in cyclohexane extract of bark. However, in comparison with our study, ethanolic extract of *S. aegyptiaca* bark contained higher amount of flavonoids 479 ± 63 mg CE/g of DM was observed in the ethanolic extract of bark. This difference may be partially explained by the fact that in our study, total flavonoids content was expressed by quercetin equivalent. According to our research, bark of analysed *Salix* genotypes contained significant amount of phenolic acids. The presence of *p*-hydroxybenzoic, vanillic, cinnamic, *p*-coumaric, ferulic and caffeic acids was reported in the genus *Salix *[1, 25]. According to work of Podłocka-Olech et al. [1] the forms of glycosides or ester derivatives are wider spread than free phenolic acids. Furthermore, the revealed presence of pyrocatechol in the bark of some willow species raises a question regarding the safety of the application of this herbal medicine, mainly the twigs [1]. On the other hand, Freischmidt et al. [26] concluded (from the *in vitro* data) that not only flavonoids and salicin derivatives, but also catechol can probably contribute to the anti-inflammatory activity of willow bark extracts.

### 3.2. Extractability of Antioxidants

**Figure 2:**
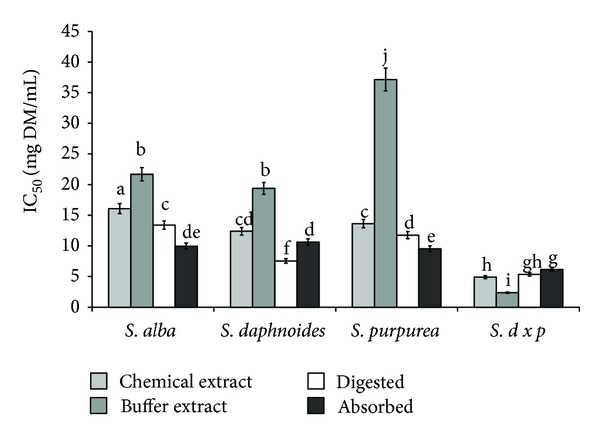
Effect of extraction system on antiradical activity of phytochemicals from bark of different *Salix* genotype. Bars (means) followed by the different letters differ significantly (Tukey-test, *P* < 0.05).

Several phenolic acids, including salicylic and caffeic acids, possess anti-inflammatory and analgesic activity which has been associated with their antioxidant activity. Scavenging oxygen free radicals decides the anti-inflammatory activity of gallic and protocatechuic acids [27]. Antioxidant activity was also shown for caffeic, ferulic and chlorogenic acids [1]. Bark of all tested *Salix *species showed significant antiradical activity (Figure 2). These properties strongly dependent of extraction system and genetic factors. Taking into account extraction procedure, better results were obtained when using EtOH than PBS buffer, with the exception of *SDP* bark. Digestion *in vitro* in all cases (except *SDP* bark) released compounds able to quench ABTS radicals. Antiradical compounds from *SA* and *SP *were easily bioavailable *in vitro*, whereas potential bioavailability of antiradical compounds released during *in vitro* digestion from bark of *SD* and *SDP* was difficult. Regardless of this, the highest antiradical activity was found for extracts obtained from *SDP* (Figure 2).

**Figure 3:**
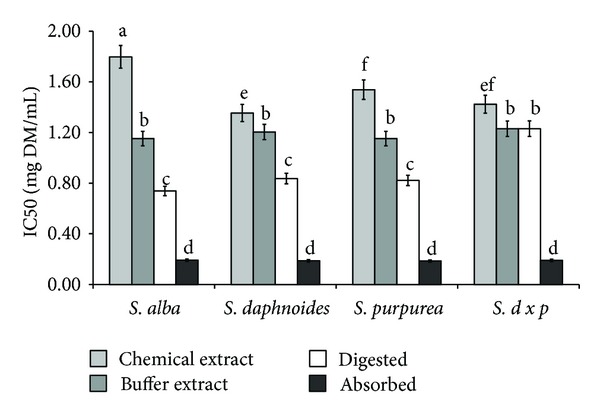
Effect of extraction system on chelating power of phytochemicals from bark of different *Salix* genotype. Bars (means) followed by the different letters differ significantly (Tukey-test, *P* < 0.05).

Taking into account the chelating power of analyzed samples the similar relationships have been received for all *Salix* varieties (Figure 3). Regardless of plant material, the lowest activity was found for chemically extractable compounds. Significantly higher activity was observed in the cases of potentially mastication-extractable compounds (buffer extracts). Digestion *in vitro* released compounds able to metal ions chelate from all samples except *SDP* bark. Most importantly, active compounds were highly bioavailable *in vitro*, whereas significant differences between samples were not found (Figure 3).

**Figure 4:**
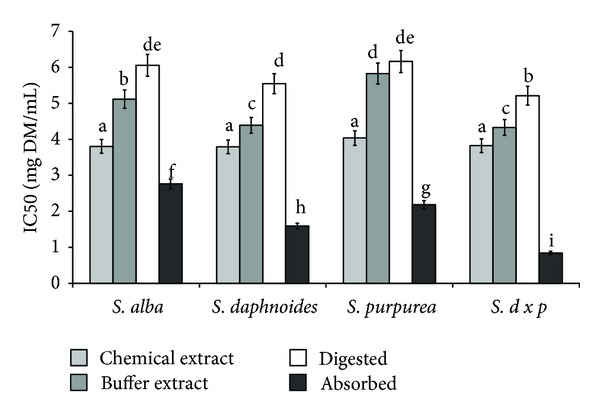
Effect of extraction system on reducing power of phytochemicals from bark of different *Salix* genotype. Bars (means) followed by the different letters differ significantly (Tukey-test, *P* < 0.05).

Bark of all *Salix* species contained ethanol-extractable compounds with comparable reducing ability. Significant differences were concerning potentially mastication-extractable phytochemicals—the highest activity was observed in the case of *SD* and *SDP* bark, whereas the lowest for bark of *SP*. Digestion *in vitro*, unexpectedly, did not release active compounds-activity of these extracts and was the lowest in all cases. Most importantly, compounds with reducing ability easily permeated dialysis membrane, which may indicate their bioavailability. In all cases activity of extracts after simulated absorption was the highest (Figure 4).

Antioxidant activity of *Salix* bark was widely studied [24, 26, 27], but comparative analysis of results was difficult due to different ways of its measure and expression. And so, antiradical activity of bark of *S. aegyptiaca* (depending on the extraction system) ranged from 10 to 105 mg quercetin equivalent/d DM [26].

The results from *in vitro* studies showed that methanolic extract from *S. nigra* bark is an effective scavenger of both superoxide and hydrogen peroxide radicals. This may be due to the phenolic content, particularly flavonoids present in *S. nigra* extract, which are believed to be potent superoxide radical scavengers. Moreover, presence of such compounds in the extract may help neutralize hydrogen peroxide into water through electron donation. The antioxidative properties of methanolic extract from *S. nigra* on scavenging hydrogen peroxide radicals were found to be superior (IC_50_ value was 175–180 *μ*g/mL) [23] while the ability to inhibit superoxide radicals was lower than that reported for *S. caprea* flower extract [28]. This activity of *Salix *bark is attributed to the high antioxidant contents which could react with free radicals to stabilize and terminate radical chain reactions [29]. Oxidative stress and inflammation have been reported to be closely associated with tumor promotion stage of carcinogenesis. The inactivation of enzymes by free radicals and the accumulation of oxidized proteins may play a critical role in the alteration of cellular function and cell death. Some essential growth regulatory proteins lose their function when damaged by free radicals [30].

**Figure 5:**
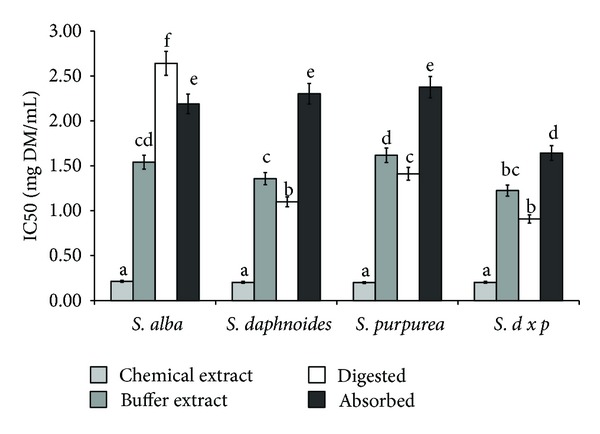
Effect of extraction system on LOX-inhibitory activity of phytochemicals from bark of different *Salix* genotype. Bars (means) followed by the different letters differ significantly (Tukey-test, *P* < 0.05).

In the light of data given in Figure 5. it may be concluded that compounds able to inhibit LOX were easily ethanol-extractable. Activity of all extracts was comparable and very high in comparison with other samples obtained from the same plant material. Significantly lower efficiency was observed after using PBS buffer. Extracts containing potentially mastication extractable phytochemicals were characterized by comparable activity. Significant differences were observed for extracts obtained after simulated digestion. Digestion *in vitro* released active compounds from all samples except *S. alba* bark. Only in this case, activity of simulated digesta was lower than activity of buffer extract. Unfortunately, compounds able to inhibit LOX with difficulty permeated dialysis membrane. Activity of extracts after simulated absorption was the lowest in all cases except *SA *sample (Figure 5).

**Figure 6:**
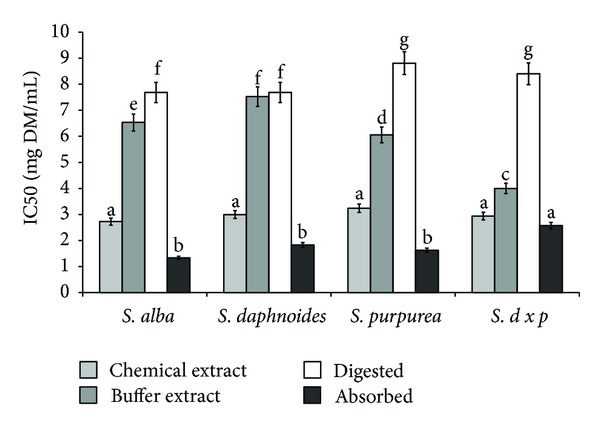
Effect of extraction system on XO-inhibitory activity of phytochemicals from bark of different *Salix* genotype. Bars (means) followed by the different letters differ significantly (Tukey-test, *P* < 0.05).

Most of the tumor promoters have been reported to increase the activity of XO thus increasing superoxide radical generation and enhancing hydrogen peroxide generation [30]. Taking into account activity of XO inhibitors, it may be concluded that the best results were obtained after using ethanol for extraction. Extractability in a buffer system was significantly lower. Especially significant differences were observed for *SA* and *SD* active compounds, whereas the lowest—for phytochemicals from *SDP*. Changes occurring during simulated digestion caused decrease of activity in all samples except *SD*. This tendency is strongly visible in *SDP* and *SP* bark. All the more interesting is the fact that XO inhibitors easily permeated dialysis membrane (Figure 6). In all cases, the highest activity was found for extracts after simulated absorption.

Results presented in this study confirm the results obtained by Sultana and Saleem [30]. Cited investigators stated that the pretreatment of *S. caprea* was observed to reverse the chemical induction in XO activity and hydrogen peroxide content. This suggests the antitumor promoting potential of *S. caprea*. High antioxidant activity under *in vitro* conditions of methanol extractfrom* S. nigra* was also confirmed. It has a great potential to ameliorate the progression of collagen-induced arthritis by controlling inflammatory proteins, nitric oxide, and antioxidant enzymes [23].

On the other hand, is interesting to note that water extract from SD bark used as elicitor significantly increased LOX and XO-inhibitory activity in broccoli sprouts. Treatment with 1% SD water extract also significantly improved SOD-like activity and increase of OH^•^ radicals scavenging ability, probably by stimulating phenolics overproduction in broccoli sprouts [31, 32].

### 3.3. Studies of Potential Bioaccessibility and Bioavailability

The gastrointestinal tract may be considered as an extractor where both the mechanical action during mastication and the chemical action during the digestive phase contribute to the extraction of phenolic compounds [33]. It should be taken into consideration that high content and activity of phytochemicals determined in chemical system not always go hand in hand in high activity *in vivo*. For this reason, the investigations of bioaccessibility and bioavailability of bioactive compounds should be carried out. Models based on human physiology have been developed as simple, cheap, and reproducible for investigating the food components *In vitro* digestion models are widely used for studying structural changes, digestibility, and release of food components under simulated gastrointestinal conditions [34].

Data given in Table 2 indicate that the best source of bioaccessible antiradical compounds was bark of *SP*, while the lowest BAC value was obtained for bark of *SDP*; however highly bioavailable *in vitro *were phytochemicals able to quench free radicals released during *in vitro* digestion from bark of *SP* and *SA*. The highest bioefficiency was found for antiradical from *SP*. Taking into account potential bioaccessibility of compounds able to metal ions chelate samples may be ordered as follows: *SA* > *SD* ≥ *SP* > *SD* *P*. Unexpectedly the highest bioavailability and bioefficiency *in vitro*  were found for SDP phytochemicals. Similar relationship was observed comparing bioavailability and bioefficiency *in vitro* of reductive compounds—the highest BAV and BEF values were determined for *SDP* bark samples while their potential bioaccessibility was relatively low. Though relatively low bioaccessibility *in vitro* (BAC values not exceed 1), LOX inhibitors released from tested *Salix* bark were strongly bioavailable and bioefficient *in vitro*. The highest values of these parameters were obtained for *S. alba *phytochemicals, whereas the lowest—for *SDP* compounds. Bark of *SA* contained also bioavailable and bioefficient compounds able to inhibit XO. Although the highest BAC value was determined in the case of *SDP* phytochemicals, potential bioavailability of these compounds was relatively low. The highest bioefficiency was determined for XO inhibitors derived from the bark of *SDP* and *SA*.

While white willow is the willow species most commonly used for medicinal purposes, purple willow (*S. purpurea*), and violet willow (*S. daphnoides*) are all salicin-rich and may be sold under the label of willow bark. The major metabolites of salicin are gentisic acid, salicylic acid, and salicyluric acid, with salicylic acid being the major component in the serum. After oral ingestion of willow bark, peak levels of salicylic acid were found in less than 2 hours. A salicin content of 240 mg corresponds to approximately 87 mg of acetylsalicylate, which is more cardioprotective than analgesic. It is also found that the bioavailability of the salicin in the formulation being evaluated was greater than that found in other studies. This suggests that different formulations of willow bark extract result in different bioavailabilities, a concept that may be applicable to all herbal medications [35].

## 4. Conclusion

Unlike in the case of synthetic pharmaceuticals based on an activity of single (chemical) active compounds, numerous phytochemical compounds act in a beneficial manner by an additive of synergistic activity in one or numerous target sites connected to physiological processes. This idea has found an application in pharmacology during investigations on combinations of few metabolites in multidirectional therapy [36]. It has been proposed that although willow extracts have been traditionally used as anti-inflammatory compounds for their salicin content, the presence of high amounts of phenolic compounds can contribute to the beneficial effects [2, 3]. Our investigations support this thesis. In the light of presented data, it may be concluded, that the best source of potentially mastication-extractable compounds with multidirectorial biological activity was SD and SDP bark. Other factors, often overlooked in studies of the biological activity of phytochemicals, are their bioaccessibility and bioavailability. Our study shows high bioaccessibility and bioavailability *in vitro* of *Salix* bark phytochemicals, which may indicate that extracts from this species of plants may provide health promoting benefits to the consumers; however, this issue requires further study.

## Figures and Tables

**Table 1:** Comparison of content and extractability factors of phenolic compounds.

	Plant material	Extracts				Extractability factors		
		Chemical	Buffer	Digested	Absorbed	MEF	DEF	AEF
TPC	*S. alba *	389.84 ± 15.32^dA^	114.64 ± 6.11^dB^	82.20 ± 2.55^dC^	51.88 ± 2.12^dD^	0.29	0.21	0.13
	*S. daphnoides *	468.97 ± 14.35^bA^	162.50 ± 5.98^bB^	141.17 ± 4.14^bC^	135.61 ± 5.54^bC^	0.35	0.30	0.29
	*S. purpurea *	414.93 ± 17.25^cA^	134.90 ± 5.12^cB^	113.29 ± 3.29^cC^	108.92 ± 4.38^cD^	0.33	0.27	0.26
	*S. d x p *	740.12 ± 20.21^aA^	249.92 ± 7.21^aB^	190.49 ± 9.22^aC^	180.74 ± 5.42^aD^	0.34	0.26	0.24

TFC	*S. alba *	21.93 ± 0.99^dA^	10.51 ± 0.55^dB^	15.48 ± 0.77^dC^	7.51 ± 0.33^dD^	0.48	0.71	0.34
	*S. daphnoides *	275.95 ± 9.58^aA^	61.73 ± 2.69^aB^	69.72 ± 1.23^aB^	31.53 ± 1.25^aC^	0.22	0.25	0.11
	*S. purpurea *	86.05 ± 3.21^cA^	27.47 ± 1.41^cB^	32.79 ± 1.13^cC^	17.04 ± 0.81^cD^	0.32	0.38	0.20
	*S. d x p *	177.90 ± 7.89^bA^	52.29 ± 2.54^bB^	58.99 ± 2.25^bB^	26.22 ± 0.84^bC^	0.29	0.33	0.15

TPA	*S. alba *	167.99 ± 6.88^dA^	210.07 ± 9.25^bB^	137.73 ± 6.55^dC^	145.19 ± 3.45^cD^	1.25	0.82	0.86
	*S. daphnoides *	252.81 ± 10.11^bA^	269.44 ± 9.13^aA^	357.61 ± 10.11^bB^	297.74 ± 9.18^bC^	1.07	1.41	1.18
	*S. purpurea *	202.24 ± 8.45^cA^	117.76 ± 7.77^cB^	210.22 ± 9.23^cC^	175.20 ± 7.13^dD^	0.58	1.04	0.87
	*S. d x p *	322.94 ± 9.48^aA^	210.40 ± 11.45^bB^	454.87 ± 12.25^aC^	433.54 ± 14.52^aD^	0.65	1.41	1.34

The values designated by the different small letters in the columns of the table (within individual extract and fraction) are significantly different (*α* = 0.05).

The values designated by the different small letters in the lines of the table are significantly different (*α* = 0.05).

**Table 2:** Comparison of antioxidants bioaccessibility (BAC), bioavailability (BAV), and bioefficiency (BEF) factors.

Activity	Plant material	Factors		
		BAC	BAV	BEF
Antiradical activity	*S. alba *	1.62	1.35	2.18
	*S. daphnoides *	2.57	0.71	1.82
	*S. purpurea *	3.16	1.23	3.90
	*S. d x p *	0.44	0.87	0.39

Chelating power	*S. alba *	1.56	3.84	5.99
	*S. daphnoides *	1.44	4.47	6.45
	*S. purpurea *	1.40	4.45	6.24
	*S. d x p *	1.00	6.48	6.47

Reducing power	*S. alba *	0.84	2.19	1.85
	*S. daphnoides *	0.79	3.49	2.76
	*S. purpurea *	0.95	2.82	2.67
	*S. d x p *	0.83	6.19	5.14

LOX inhibitory activity	*S. alba *	0.85	5.77	4.91
	*S. daphnoides *	0.98	4.20	4.11
	*S. purpurea *	0.69	5.44	3.74
	*S. d x p *	0.48	3.27	1.56

XO inhibitory activity	*S. alba *	0.58	1.21	0.70
	*S. daphnoides *	1.24	0.48	0.59
	*S. purpurea *	1.15	0.59	0.68
	*S. d x p *	1.35	0.55	0.75
